# The First Cavernicolous Species of *Arrhopalites* (Collembola, Symphypleona, Arrhopalitidae) from China and Its Phylogenetic Position †

**DOI:** 10.3390/insects16030314

**Published:** 2025-03-18

**Authors:** Nerivania Nunes Godeiro, Yun Bu, Gleyce da Silva Medeiros, Yan Gao, Robert S. Vargovitsh

**Affiliations:** 1 Shanghai Natural History Museum, Shanghai Science & Technology Museum, Shanghai 200041, China; nerivania@gmail.com (N.N.G.); gaoy@sstm.org.cn (Y.G.); 2 Laboratório de Collembola, Departamento de Botânica e Zoologia, Centro de Biociências, Universidade Federal do Rio Grande do Norte (UFRN), Natal 59072-970, RN, Brazil; gleycemedeiros96@gmail.com; 3 Schmalhausen Institute of Zoology, National Academy of Sciences of Ukraine, Bohdan Khmelnitsky Str. 15, 01030 Kyiv, Ukraine

**Keywords:** Asia, cave, mitochondrial DNA, phylogeny, springtails, taxonomy

## Abstract

*Arrhopalites* Börner, 1906 includes 40 valid species, with 20 considered troglobionts, exclusive to caves. *Arrhopalites beijingensis* sp. nov. is the third species from China and the first from a cave habitat, collected in the Xianrendong Cave, Beijing, Junzhuang Town. It resembles several species of the *Caecus* group, possessing spine-like chaetae on the head, five to seven subsegments on Ant IV with annulated separations, and lacking cuticular spines on Abd VI; it differs from these species in its various characteristics. The mitogenome of *A. beijingensis* sp. nov. was assembled (14,774 bp in length), marking the first made available from the genus. Due to the incipient number of published mitochondrial genomes of Symphypleona, we downloaded raw sequencing data and performed the assemblies and annotations of 11 species. Our phylogenetic analyses comprised 24 mitogenomes in total; of those, 22 were of Symphypleona and 2 were of outgroups. *A. beijingensis* sp. nov. was placed closer to *Pygmarrhopalites spinosus* (Rusek, 1967), with high support. Arrhopalitidae was found as a sister group to Katiannidae, both part of Katiannoidea. The two species of Sminthuridida were placed as a sister group to all other Appendiciphora. More systematic analyses and enhanced sampling from key lineages are needed to better understand Symphypleona’s internal relationships and evolution.

## Simple Summary

*Arrhopalites beijingensis* sp. nov., collected from Xianrendong Cave, Beijing, China, is described. Detailed illustrations of morphological features are provided, along with comparative remarks highlighting similarities and differences between the new species and other members of the *caecus* species group. The mitochondrial genome of the new species was characterized and used in a phylogenetic analysis that included 21 other Symphypleona species and two outgroups. This represents the most comprehensive phylogenetic analysis of the group to date; however, due to limited support values at deeper nodes, conclusions regarding families relationships were avoided.

## 1. Introduction

Although several research groups have recently made efforts to characterize and study the systematics of the local Collembola fauna, our understanding of the Chinese Symphypleona is still very limited. Only 63 species of the order have been found in China to date, distributed in six families and 24 genera [1]. Most of the descriptions are outdated, from more than 20 years ago, and with the decreasing number of taxonomists, the morphological study of the group is threatened. Concerning the genus *Arrhopalites* [2] (Arrhopalitidae), only two species were recorded: *A. brevicornis* Godeiro, Zhang & Bellini, 2022, collected at Jilin Province in a transitional zone of cropping and grazing [3], and *A. pukouensis Wu & Christiansen*, 1997, collected at Jiangsu Province in the first layer of litter from an urban mountain [4].

The knowledge about the phylogenetic position of Arrhopalitidae is still incipient. The only study including the group was made by D’Haese [5]. He used one species of *Pygmarrhopalites* (*P. sericus* (Gisin, 1947)—Arrhopalitidae) and one of *Sminthurinus* (*S. bimaculatus* Axelson, 1902—Katiannidae) in his analyses, which contained a well-represented dataset of Collembola species. His result was based on the D1 and D2 regions of 28S rDNA, and he discovered the two families were sister groups, in agreement with the morphology. Concerning the order Symphypleona, recent studies have divergent results regarding its phylogenetic relationships. Bellini et al. [6] used 124 mitogenomes of Collembola, 9 of them Symphypleona, and proposed Neelipleona + (Symphypleona + (Entomobryomorpha + Poduromorpha)). Yu et al. [7], using 45 draft genomes, 8 of them Symphypleona, suggested Entomobryomorpha + (Symphypleona + (Neelipleona + Poduromorpha)). From these results, we can resume that the correct sister relationship of Symphypleona is still unclear.

In this paper, we describe a new species of the *A. caecus* (Tullberg, 1871) group of species *sensu* Vargovitsh [8]. This is the first cavernicolous species of *Arrhopalites* recorded in China. We also characterize its mitochondrial DNA, which is the first to be available from this genus, and present a phylogeny of Symphypleona to test the phylogenetic placement of the newly sequenced species.

## 2. Materials and Methods

### 2.1. Samples Collection

**Figure 1:**
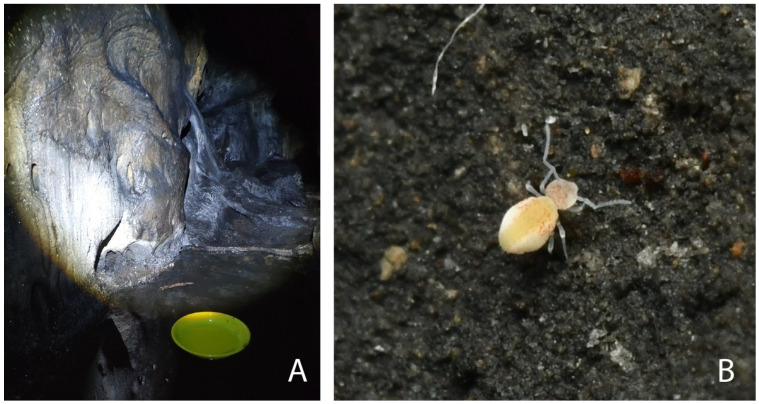
(**A**) Floating yellow plate trap (YPT) used to collect the specimens. (**B**) Individual of *Arrhopalites beijingensis* sp. nov. in natural habitat (photos are courtesy of Yang HC and Zhou DK).

The new species was collected by Yang HC and Zhou DK in November 2023 in Beijing Province, Xianrendong Cave (depth 30–40 m), using floating plates upon the water surface or directly from the cave walls using entomological aspirators (Figure 1). The collection site is 150 m from the entrance of the cave. *Papirioides caishijiensis* Wu & Chen, 1996 and *Sminthurinus bimaculatus* specimens were collected by Godeiro NN, Bu Y, and Gao Y in November 2023 in Yunnan Province, Dali City, using an entomological aspirator. The specimens were kept in absolute ethanol and frozen.

### 2.2. Taxonomic Identification and Description

A stereomicroscope Teelen XTL-207 (Shanghai Dilun Optical Instrument Co., Ltd., Shanghai, China) was used to bleach and diaphanize the specimens, first in 5% KOH and then 10% lactophenol for three minutes/each. Specimens were placed in a slide on a drop of Hoyer’s liquid and covered by a glass coverslip. Slides were dried in an oven at 50 °C for 10 days [9]. A Leica DM2500 microscope (Leica Microsystems, Wetzlar, Germany) was used to visualize the structures and further identify the specimens of *P. caishijiensis* and *S. bimaculatus* using specialized literature and identification keys [10,11,12]. Illustrations were made with a drawing tube attached to the microscope; they were posteriorly vectorized with CorelDRAW 2024 v25. The habitus of the species was photographed in 70% ethanol under a Leica S8APO (Leica Microsystems, Wetzlar, Germany) stereomicroscope attached to a Leica DMC4500 camera (Leica Microsystems, Wetzlar, Germany), using Leica Application Suite software v. 3.7 (Leica Microsystems, Wetzlar, Germany).

Chaetotaxy nomenclatures are used following Betsch and Waller [13] for the head, Fjellberg [14] for the mouthparts, Vargovitsh [15,16] for the great abdomen, Bretfeld [17] for the fifth abdominal segment and anterior part of the great abdomen (Th II–Abd I), Betsch [18] for the sixth abdominal segment, and Nayrolles [19,20,21] for the appendages.

### 2.3. DNA Isolation and Sequencing

Shanghai Yaoen Biotechnology Co., Ltd., Shanghai, China, was responsible for all laboratory tests, from DNA isolation to sequencing. The experiments were conducted in accordance with the guidelines provided by the kit manufacturers. Only one individual of the new species as well as *P. caishijiensis* and *S. bimaculatus* were used for DNA extraction. TIANamp MicroDNA extraction kit (Tiangen Co., Ltd., Beijing, China) was utilized to extract DNA and KAPA Hyper Prep Kit (Roche, Basel, Switzerland) was used to build the libraries. An Illumina NovaSeq 6000 platform produced approximately 10 Gbp of paired-end reads from each species, each 150 bp in length.

### 2.4. Mitogenomes Assemblies and Annotations

Before performing the main analyses, a quality control of the raw sequencing data was performed to remove duplicates and low-quality regions and to normalize and correct possible errors. These steps were performed using a custom pipeline and BBTools (sourceforge.net/projects/bbmap/ accessed on 13 March 2025). MitoZ v. 3.6 [22] was used to assemble, annotate, and visualize the mitogenomes of the new species as well as *P. caishijiensis*, *S. bimaculatus*, and the other 13 species data downloaded from the SRA database (NCBI). The following tools were utilized by MitoZ during these processes: Fastp v. 0.23.4 [23] for filtering, SPAdes v. 3.15.5 for assembly, HMMER v. 3.4 [24] for searching homologous sequences and making alignments, BLAST+ v. 2.16.0 [25], GeneWise v. 2.2.0 [26], Infernal v. 1.1.5 [27], and MiTFi [28] for annotation; Circos v. 0.69 [29], BWA v. 0.7.18 [30], SAMtools v. 1.15.1 [31], and ETE3 toolkit v. 3.1.3 [32] were used to visualize the mitogenomes and draw the sequencing coverage distribution track.

The three newly sequenced mitogenomes and their raw sequencing data were submitted to the National Center for Biotechnology Information (NCBI), nucleotide and Sequence Read Archives (SRA) databases; the accession numbers are listed at the end of this manuscript. The mitogenomes sequences assembled from the published data retrieved from the SRA database were deposited to Figshare (https://doi.org/10.6084/m9.figshare.26693296 accessed on 20 December 2024), and taxonomic information, country, accession number, and the references of the source data from all analyzed species are listed on Table 1.

### 2.5. Matrix Generation and Phylogenetic Analyses

To investigate the phylogenetic placement of the three new mitogenomes within the Symphypleona order, we included 19 additional Symphypleona species, representing six major families. *Homidia socia* Denis, 1929 (Entomobryomorpha) and *Brachistomella parvula* (Poduromorpha) were used as outgroups. A phylogenetic matrix containing 24 species was generated following a custom pipeline. In summary, all 13 Protein Coding Genes (PCG’s) from each species were placed in different folders. TransDecoder v. 5.5.0 (http://transdecoder.github.io/ accessed on 05 September 2024) was used to translate the nucleotides into amino acids. MAFFT v. 7.470 with “L-INS-I” algorithm was used to align and Trimal v. 1.4.1 [42] trimmed the alignments with the “-gappyout” option. FASconCAT-G v.1.04 [43] concatenated the final sequences into one matrix with 3530 amino acid sites (2191 parsimony informative sites). IQ-TREE v.2 [44] was used to perform the maximum likelihood (ML) analyses, with a partitioned dataset. ModelFinder [45] was invoked to choose the best substitution model for each partition; the details of the models used are presented in Table S1. A total of 1000 SH-aLRT and UFBoot2 [46] replicates were run for ML analyses. Bayesian analyses were performed in PhyloBayes-MPI v. 1.8 [47], using the CAT + GTR site-heterogeneous mixture model. Two independent Markov chain Monte Carlo chains (MCMC) were run and terminated when the two runs adequately converged (maxdiff < 0.1). A total of 10% of the generated trees was removed as burn-in, and a consensus tree was calculated from the remaining trees. Generated trees were visualized with FigTree v1.4.2 (available on https://tree.bio.ed.ac.uk/software/figtree/ accessed on 15 September 2024).

## 3. Results

### 3.1. Taxonomy

**Figure 2:**
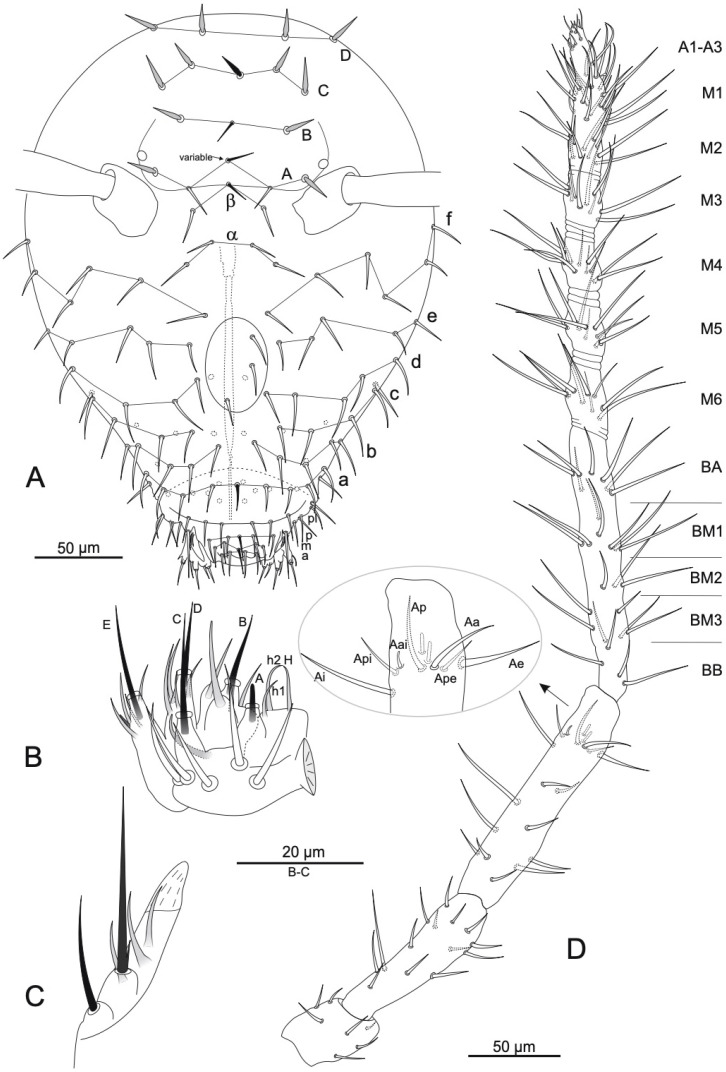
*Arrhopalites beijingensis* sp. nov. (**A**) Chaetotaxy of head, frontal view; (**B**) chaetotaxy of labial palp; (**C**) maxillary outer lobe; (**D**) antenna (detail shows the sensory organ of Ant III). Nomenclatures of chaetae in (**A**): after [13], (**B**): [14], (**D**): [21].

**Figure 3:**
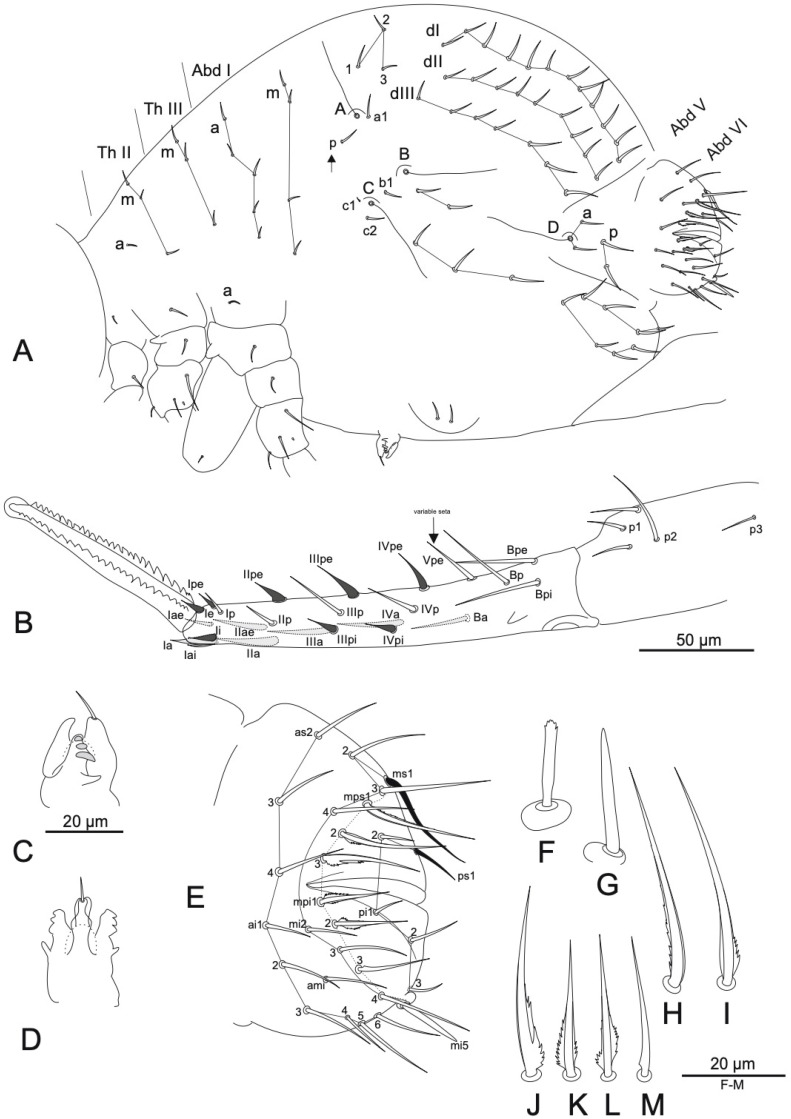
*Arrhopalites beijingensis* sp. nov. (**A**) Chaetotaxy of great abdomen, lateral view, the arrow denotes the position of seta p above trichobothrium B; (**B**) mucro and dens, dorso-lateral view; (**C**) tenaculum, lateral view (**D**) tenaculum, dorsal view; (**E**) chaetotaxy of female Abd VI, lateral view; (**F**) subanal appendage (=mi5), dorsal view; (**G**) subanal appendage, lateral view; (**H**) mps1; (**I**) mps2; (**J**) mps3; (**K**) mpi1; (**L**) mpi2; (**M**) mpi3. Nomenclatures of chaetae in (**A**): after [15,16], (**B**): [20], (**E**–**M**): [18].

**Figure 4:**
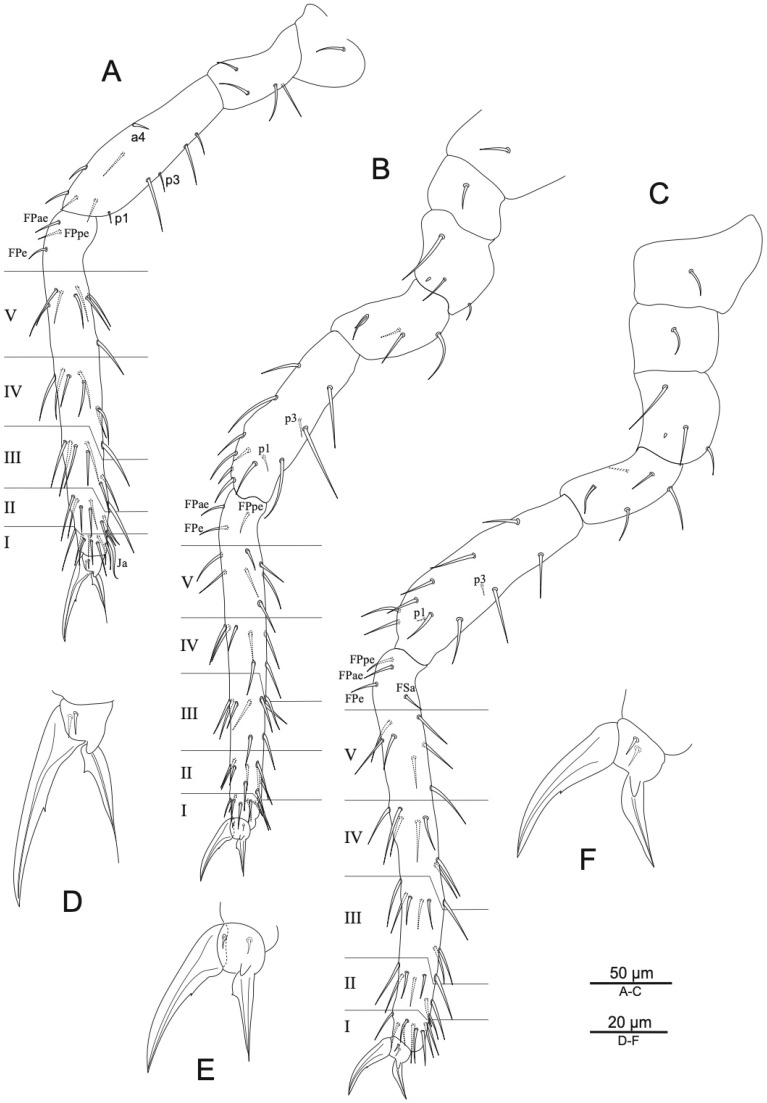
*Arrhopalites beijingensis* sp. nov. chaetotaxy of legs, anterior view: (**A**) foreleg; (**B**) mid-leg; (**C**) hind leg; (**D**) foreleg foot complex; (**E**) mid-leg foot complex; (**F**) hind leg foot complex. Nomenclatures of chaetae in (**A**–**C**): after [19].

Family Arrhopalitidae Stach, 1956;

Genus *Arrhopalites* Börner, 1906;

*Arrhopalites beijingensis* Godeiro & Vargovitsh sp. nov.;

(Figure 2, Figure 3 and Figure 4, Table 2 and Table 3).

Diagnosis. Antenna about 2 times length of head; Ant IV with 14 whorls of chaetae and with 6 subsegments indistinctly separated from each other by 1–3 annuli. Head dorsum with 13 distinctly spine-like chaetae. Trichobothria ABC form a right angle and AB > BC. Sixth abdominal segment without cuticular spines; circumanal chaetae thickened, some of them lamellated and basally serrated; subanal appendage rod-like, apically serrated. All unguis slender, without tunica, with very small inner tooth. Tip of unguiculus I–III does not reach tip of corresponding unguis; unguiculus I–II both with and III without corner tooth. Tenaculum with 1 chaeta. Manubrium with 5 + 5 chaetae. Dens with 3, 2, 1, 1, 1 thick anterior chaetae, **Ia** spine-like. Posterior side with chaetae **Ii**, **Ie**, **Ipe**–**IVpe** spine-like. Tip of mucro swelled.

Type material. Holotype on slide “SYBE-01”: female, China, Beijing Province, Junzhuang Town, Huiyu Village, in the aphotic zone of Xianrendong Cave 40.02198 N 116.09009 E, Alt. 297 m, 23.XI.2023, Yang HC and Zhou DK leg. Paratypes on slides: 6 females, same data as for holotype. Holotype and 4 paratypes on slides are deposited in the collection of the Shanghai Natural History Museum (SNHM), China, and 2 paratypes on slides are kept in the collection of Schmalhausen Institute of Zoology, National Academy of Sciences of Ukraine, Kyiv. In addition to slides, three specimens are kept in alcohol at SNHM.

Description. Female: body length (excluding antennae and furca) about 1 mm, whole body with light yellow background and orange spots of dorsal pigmentation on lateral body and head (Figure 1B). Measurements of type specimens are given in Table 2, and length ratios between selected structures are provided in Table 3.

Antenna (Figure 2D): length about 2 times length of head. Mean length ratio of antennal segments I:II:III:IV = 1:2.3:3.3:9. (1:2.3:3.3:10 in holotype) (Table 3). Ant IV indistinctly subdivided into 6 (sometimes 5 or 7) subsegments, separated from each other by 1–3 annuli (sometimes hardly visible). Subsegmental formula depends on number of subsegments and if 6 subsegments present, then formula is as follows: 1 + 4 + 1 = (A + M1–M2) + (M3–M6) + (B). Ant IV bears 14 whorls of chaetae. Ant III has 18 chaetae and 2-rods as sensory organ; **Aai** is a short sensillum, and **Api** and **Ape** are thinner and smaller than others. Ant II with 14 chaetae. Ant I has 7 chaetae, of which posterior subapical one is microchaeta.

Head (Figure 2A). Eyes 1 + 1, small (~7.5 µm in diameter), unpigmented. Dorsal area **A**, **B**, **C**, **D** series with 2(+1 axial)/1(+1 axial)/2(+1 axial)/2 chaetae, respectively; 13 chaetae are spine-like with broadened sockets (in row **A**/**B**/**C**/**D**: 1/1/2(+1 axial)/2, respectively); other chaetae are not modified. Interantennal area **α** and **β** series with 2/1(+1 axial) chaetae, respectively. Clypeal area **a**, **b**, **c**, **d**, **e**, **f** series with 4(+1 axial)/5/5/5/5/6 chaetae, respectively, and three medial asymmetrical chaetae. Labral/prelabral chaetotaxy following the formula **a**/**m**/**p**/**pl**: 4/5/5/6. Labial basomedian field with four chaetae, basolateral field with five chaetae. Maxillary outer lobe (Figure 2C) with basal chaeta smaller than apical, apical chaeta with a basal tooth; sublobal plate with three chaeta-like appendages (sublobal hairs). Maxillae typical, with six lamellae; the three internal ones are serrated. Labial palp (Figure 2B) with five proximal chaetae, five papillae with long deeply embedded terminal sensilla and associated guard chaetae, and 3 hypostomal chaetae. Formula of guard chaetae of each papilla is as follows: **H**(2)/**A**(0)/**B**(4)/**C**(0)/**D**(4)/**E**(4 + lateral process). Mandibles with 4–5 incisive apical teeth.

Large abdomen (Figure 3A): Th II and III each with sensillum **a** and 3 **m** chaetae; Abd I has 5 **a**, 4 **m** and 1 **p** chaetae. Trichobothrial complex: **ABC** form around right angle and **AB** is about 1.5 times longer than **BC**; chaeta **b1** lies in line **BC**, closer to **C**. Chaeta **c1 is** near trichobothrium **C** as microchaeta (~5 μm). Posterior lateral complex with 5 chaetae in two rows (2 + 3); furca base complex with 8 chaetae in two rows (4 + 4); central dorsal complex with 3 chaetae; posterior dorsal complex with about 28 chaetae arranged in 3 longitudinal rows **dI**, **dII**, **dIII** with 12/9/7 chaetae, respectively. Ventral complex with 1 or 2 chaetae.

Fifth abdominal segment (Figure 3A) with 2 chaetae and trichobothrium **D** in row **a**, and 2 chaetae in row **p**. Genital field with 2 + 2 or 3 + 3 chaetae close to anterior margin of genital opening.

Sixth abdominal segment (Figure 3E): cuticular spines absent. Dorsal anal valve with 12 chaetae per side (10 + 2 axial), **ms5** absent; each of lateral valves have 19 chaetae. Circumanal chaetae (dotted row in Figure 3E) broadened; **mps1**–**mps3** and **mpi1**–**mpi2** more or less lamellated and basally serrated (Figure 3E–M). Subanal appendage (**mi5**): rod-like, with weak apical serration (Figure 3F) (pointed in lateral orientation, as in Figure 3G), inserted into globular papilla. In total, normally 60 chaetae on Abd VI are present (10 + 2 axial + 10) + (19 + 19).

Ventral tube with 1 + 1 subapical microchaetae. Tenaculum (Figure 3C,D): each ramus with 3 teeth and basal process; anterior lobe with 1 apical chaeta; tip of posterior lobe is approximately on same level with tip of anterior.

Leg I (Figure 4A,D): Epicoxa, subcoxa, and coxa with 1/0/1 chaetae, respectively. Trochanter with 4 chaetae; femur with 11 chaetae, **a4** turned perpendicularly to the longitudinal axis of the segment. Tibiotarsus with 44 chaetae, whorls I–V with 9/8/8/8/8 chaetae, respectively, **Ja** curved and thickened, region **F** with 3 primary **FP** chaetae (**e**, **ae**, **pe**). Pretarsus with 1 anterior and 1 posterior microchaetae. Foot complex (Figure 4D). Unguis: narrowed and slender, without tunica, with small inner tooth, about 4 times shorter than tibiotarsus. Unguiculus: thin, with corner tooth, apical filament short, not reaching tip of unguis.

Leg II (Figure 4B,E): Epicoxa, subcoxa, and coxa with 1/1/3 chaetae, respectively; coxa with spiny microsensillum. Trochanter with 3 ordinary chaetae and anterior trochanteral organ. Femur with 13 chaetae, 2 posterior ones (**p1** and **p3**) are shorter and thinner than others. Tibiotarsus with 43 chaetae, whorls I–V with 9/8/8/8/7 chaetae, respectively, region **F** with 3 **FP** chaetae (**e**, **ae**, **pe**). Foot complex (Figure 4E). Unguis: broader than in leg I, without tunica, with very small inner tooth, about 4–4.5 times shorter than tibiotarsus. Unguiculus: broader than in leg I, with corner tooth and short apical filament, not reaching tip of unguis.

Leg III (Figure 4C,F): Epicoxa, subcoxa, and coxa with 1/1/3 chaetae, respectively; coxa with spiny microsensillum. Trochanter with 4 ordinary chaetae and trochanteral organ; femur with 12 chaetae, 2 posterior ones (**p1** and **p3**) as microchaetae. Tibiotarsus longer than in legs I–II, with 44 chaetae, whorls I–V with 9/8/8/8/7 chaetae, respectively; region **F** with 3 primary **FP** chaetae (**e**, **ae**, **pe**) and secondary **FSa** chaeta. Foot complex (Figure 4F). Unguis: broader than in leg II, without tunica, with small inner tooth, about 5–6 times shorter than tibiotarsus. Unguiculus: broad, shorter than unguis, corner tooth absent; apical filament not developed.

Furca (Figure 3B): manubrium with 5 + 5 dorsal chaetae, **p3** thin and short. Dens with 16(15) posterior chaetae, **Vpe** often absent, **Ii**, **Ie**, **Ipe**–**IVpe** spine-like; anterior side with 3, 2, 1, 1, 1 thick chaetae from apex to basis, **Ia** distinctly spine-like. Mucro (Figure 3B): posterior lamellae with 20–29 teeth each, anterior lamella weakly developed, tip broadened and globular. Dens approximately 1.7 times as long as mucro.

Male: not seen.

Variability. Body size of adults vary considerably, ranging from 0.7 to 1.3 mm (see Table 2 and Table 3 for measurements). Antenna in one specimen shorter than in others, at 1.5 times size of head. Two specimens possess only 5 subsegments and 12 whorls of chaetae in Ant IV instead of 14. Some specimens possess 7 subsegments but still have 14 whorls of chaetae. Axial chaeta of row **A** on head dorsum absent in specimen with shorter antenna. Femur I in one specimen with 12 chaetae instead of 11. Chaeta **Vpe** often absent on one or both (left and right) dens. Chaeta **ms2** of Abd VI in one specimen asymmetrically absent on one side. Lateral valve in one specimen with 17 chaetae instead of 19.

Ecology and distribution. *Arrhopalites beijingensis* sp. nov. was collected 150 m from the entrance of Xianrendong Cave, a limestone cave with a depth of 30–40 m. The cave is located in Beijing Province, which has a cold, temperate climate with increased summer rainfall. Under Köppen classification [48], this climate is Dwa (humid continental). The mean annual temperature ranges from −5 °C to +27 °C.

Etymology. The epithet “*beijingensis*” refers to Beijing City, the capital city of China, where the type specimens were collected.

Remarks. *Arrhopalites beijingensis* sp. nov. belongs to the *caecus* group of species with 3, 2, 1, 1, 1 anterior dens chaetae [8] and can be compared with species of this group possessing spine-like chaetae on head, five to seven subsegments on Ant IV with annulated separations, and a lack cuticular spines on Abd VI. Three species share this combination of traits: *A. gul* Yosii, 1966 from South Korea; *A. peculiaris* Vargovitsh, 2009 from Crimea (Ukraine); and *A. abchasicus* Vargovitsh, 2019, from the Caucasus (the presence of cuticular spines on Abd IV in this species varies from 0 to 4). The new species differs from the highly troglomorphic *A. gul* in several respects: a shorter antennae (2 times vs. 2.9 times head length), a normal (vs. elongated) unguis shape, the presence of a corner tooth on the unguiculus of legs I and II (absent in *A. gul*), several spine-like chaetae on the dens (vs. 1 in *A. gul*), and 12 chaetae per side on the dorsal anal valve (vs. 13 in *A. gul*). It can be distinguished from *A. peculiaris* by the presence of 13 spine-like chaetae on the head dorsum (vs. 5 in *A. peculiaris*), shorter antennae (2 times vs. 2.4 times head length in *A. peculiaris*), more numerous spiny chaetae on the dens, the shape of its circumanal chaetae (broadened and serrated vs. thin and smooth in *A. peculiaris*), and the number of chaetae on the dorsal valve of Abd VI (12 per side vs. 11 in *A. peculiaris*). Finally, the new species differs from *A. abchasicus* in the number of spine-like chaetae on the head dorsum (13 vs. 9 in *A. abchasicus*), the shape of Ant III (not basally swollen vs. basally swollen in *A. abchasicus*), the presence of an inner tooth on unguis III (absent in *A. abchasicus*), and the absence of a corner tooth on the unguiculus III (present in *A. abchasicus*).

### 3.2. Mitochondrial Genomes

Newly generated mitogenomes of *Arrhopalites beijingensis* sp. nov., *Papirioides caishijiensis*, and *Sminthurinus bimaculatus* have a length of 14,774, 15,271, and 14,922 base pairs, respectively (Figures S1–S3). We analyzed the gene order of all the 22 mitogenomes of Symphypleona that were automatically annotated and that we used in the phylogenetic analyses; from these, only 7 mitogenomes (*Bourletiella arvalis* (Fitch, 1863), *B. hortensis* (Fitch, 1863), *Deuterosminthurus bicinctus* (Koch, 1840), *Dicyrtomina saundersi* (Lubbock, 1862), *Ptenothrix huangshanensis* (Chen & Christiansen, 1996), *Sminthurides aquaticus* (Bourlet, 1842), and *Sminthurides bifidus* (Mills, 1934) presented the same gene order as the ancestral Pancrustacean arrangement, which is the commonest in Collembola [38]. Other sequences have small differences on the transfers RNAs (tRNAs) when compared with the ancestral gene order; most of them are transversions or inversions. We also observed many missing tRNAs, but we did not consider this as a difference because it can be caused by an annotation omission. Concerning the PCG’s and ribosomal RNA genes, all mitogenomes followed the Pancrustacean arrangement which is ND2–COX1–COX2–ATP8–ATP6–COX3–ND3–ND5–ND4–ND4L–ND6–CYTB–ND1–16s-RNA–12s-RNA.

Regarding *Arrhopalites beijingensis* sp. nov., the coverage of the mitochondrial reads was low. We believe that the family Arrhopalitidae has a high divergence, because the assembly of *Pygmarrhopalites spinosus* (produced using NCBI-SRA data) was also not good. Both species were assembled using the same amount of data (10Gbp) used for other species of Collembola which aimed at obtaining good results. The control region (CR) located between the 12s ribosomal RNA and the tRNA-Ile (Isoleucine) was not recovered. Instead, a large non-coding region between tRNA-Ser (Serine) and ND1 of 604 bp was found, and a similar abnormality was present in the mitogenome of the Antarctic springtail *Cryptopygus antarcticus* Willem, 1901, but with a smaller length (123 bp) [41]. Despite the low coverage, all protein coding genes were correctly annotated in the mitogenome of *Arrhopalites beijingensis* sp. nov., but three tRNAs were absent (tRNA-Ile, tRNA-Gln, and tRNA-Cys) (Figure S1, Table S2).

In the *Papirioides caishijiensis* sequence, one transversion occurred in the tRNA-Ile (Isoleucine); also, two tRNA deletions of the tRNA-Leu (Leucine) and tRNA-Cys (Cysteine) were verified (Figure S2, Table S3).

An inversion occurred between the tRNA-Ala (Alanine) and tRNA-Arg (Arginine) in the *Sminthurinus bimaculatus* mitogenome (Figure S3, Table S4) from our representative of the Chinese population; the same inversion was identified in the mitochondrial DNA of all the other four sampled representatives of Katiannidae. We also observed that the four sampled Sminthuridae presented a transversion of tRNA-Asp (Acid aspartic), which, in the ancestral gene order, comes before ATP8, and in this family, comes before ND1. No more obvious characteristics related to gene order were observed for the other families studied here.

### 3.3. Phylogenetic Placement of the New Mitogenomes

**Figure 5:**
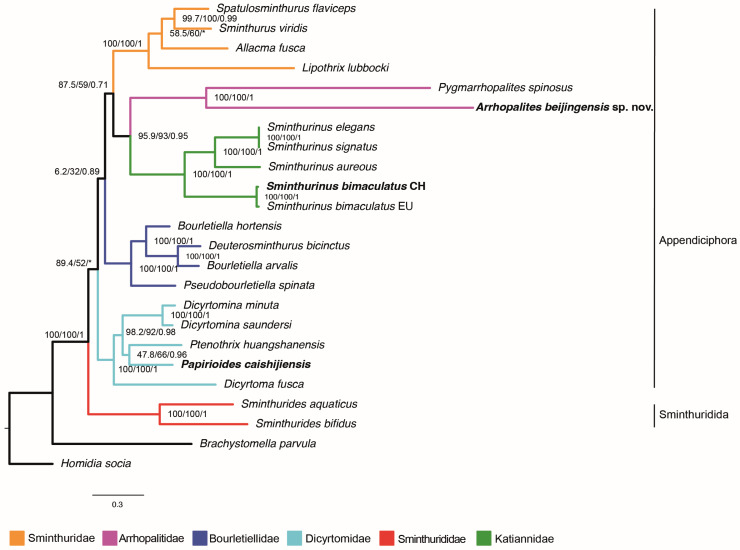
Phylogenetic placement of *Arrhopalites beijingensis* sp. nov., *Papirioides caishijiensis*, and *Sminthurinus bimaculatus*. The numbers at the nodes represent the SH-aLRT support, the bootstrap values (both for maximum likelihood), and the posterior probability (for Bayesian inference support), respectively. ‘*’ represents a discordant branch in the Bayesian inference result.

Our maximum likelihood and Bayesian analyses, including 22 species of Symplypleona and based on 13 mitochondrial genes, recovered the three newly sequenced mitogenomes correctly placed according to their taxonomy (Figure 5). *Arrhopalites beijingensis* sp. nov. was grouped with *Pygmarrhopalites spinosus* with absolute support, both belonging to the family Arrhopalitidae. *Papirioides caishijiensis* was grouped with *Ptenothrix huangshanensis* with moderate support (SH-aLRT = 47.8; Bootstrap = 66; pp = 0.98), both belonging to the subfamily Ptenothricinae. Lastly, the Chinese representative of *Sminthurinus bimaculatus* was grouped with the European representative of the same species, in a basal branch to the other three sampled species of *Sminthurinus* with high support.

Concerning the monophyly, all six sampled families were recovered as monophyletic with strong support; Sminthurididae (suborder Sminthuridida sensu Sánchez-García and Engel [49] was recovered as the sister group of the suborder Appendiciphora sensu Bretfeld [50], but with a low support value in the ML tree (SH-aLRT = 89.4/Bootstrap = 52) and in the Bayesian inference. The result was undefined, with a trichotomy of Sminthurididae + Dicyrtomidae + other taxa.

Regarding subfamilies, the two sampled Ptenothricinae were placed in an internal branch of Dicyrtominae, but with low support value (SH-aLRT = 47.8; Bootstrap = 66; pp = 0.96). The subfamily status of Parabourletiellinae was not recovered, with its representative clustered with species of Bourletiellinae. The subfamily Sminthurinae was recovered as monophyletic with high support values.

## 4. Discussion

Despite being the most representative phylogenetic study of Symplypleona to date, the focus of the present article was not on resolving its internal family’s relationships. Our goals were to characterize the newly sequenced mitogenomes, validate their phylogenetic placement, expand the genetic resources for future phylogenetic research, and describe a new species of *Arrhopalites*. Therefore, we will refrain from discussing our results regarding the phylogeny of the families.

*Arrhopalites beijingensis* sp. nov. is the first cavernicolous species of this genus described in China, it is weakly to moderately troglomorphic, having somewhat elongated antennae. Until now, only three *Arrhopalites* species present distinct progressive troglomorphisms (*A. gul*, Yosii, 1966 from South Korea, *A. macronyx* Vargovitsh, 2012, and *A. profundus* Vargovitsh, 2022 from the Caucasus), such as tangible elongated antennae, remarkably elongated unguis, an enlarged sensory organ of the third antennal segment, and other characteristics [51]. Based on the anterior dens chaetotaxy, the new species belongs to the *caecus* species group. In Asia, eleven other species within this group have been described, including two from China: *A. pukouensis* Wu & Christiansen, 1997 from Jiangsu Province in the east, and *A. brevicornis* from Jilin Province in the northeast. *Arrhopalites beijingensis* sp. nov. differs from both by the shape of its circummanal chaetae in the small abdomen, its longer antennae, and the presence of subsegments on the Ant IV. *A. pukouensis* lacks eyes and body pigment, whereas *A. brevicornis* and *A. beijingensis* sp. nov. both have 1 + 1 eyes and orangish spots distributed in the dorsal part of the body. Additionally, unlike the other two, *A. brevicornis* possesses 4 + 4 cuticular spines on Abd VI.

The gene order of the newly sequenced *A. beijingensis* sp. nov., *Papirioides caishijiensis*, and *Sminthurinus bimaculatus* is different from the most common organization found in Collembola mitochondrial genomes, the ancestral gene order (AGO) for Pancrustacea. In our study, we observed that only 7 out of 22 sampled species of Symphypleona present with the AGO. Leo et al. [38] and Cucini et al. [52] also observed this divergence in Symphypleona species. Leo et al. [38] suggested that gene order data can offer compelling evidence for the monophyly of a group when multiple sequences exhibit a shared derived gene order. Our study confirmed that sampled members of Katiannidae and Sminthuridae share the same gene order alteration, which was not observed in other families. The variations in mitochondrial gene arrangement among the 22 Symphypleona species studied here raise questions about the gene rearrangements process and mitochondrial genome evolution. These questions can be more effectively addressed as more complete mitochondrial genome sequences become available for this group.

## Figures and Tables

**Table 1:** Taxonomical information, country, and NCBI accession numbers of all samples used for phylogenetic analyses. Newly sequenced and assembled mitogenomes are marked in bold. ND—not defined; NA—not available; NP—not published.

	Species	Order	Suborder	Superfamily	Family	Subfamily	NCBI Number	Country	Source
1	*Homidia socia*	Entomobryomorpha	ND	Entomobryoidea	Entomobryidae	Entomobryinae	MN480464.1	China	Wu and Chen [33]
2	*Brachistomella parvula*	Poduromorpha	ND	Neanuroidea	Brachistomellidae	ND	MN660050.1	China	Jiang et al. [34]
3	*Allacma fusca*	Symphypleona	Appendiciphora	Sminthuroidea	Sminthuridae	Sminthurinae	MT547779.1	Italy	Nardi et al. [35]
4	***Arrhopalites beijingensis*** sp. nov.	**Symphypleona**	**Appendiciphora**	**Katiannoidea**	**Arrhopalitidae**	**ND**	**PQ046244.1**	**China**	**This study**
5	*Pygmarrhopalites spinosus*	Symphypleona	Appendiciphora	Katiannoidea	Arrhopalitidae	ND	SRR17308015	NA	Collins et al. [36]
6	*Bourletiella arvalis*	Symphypleona	Appendiciphora	Sminthuroidea	Bourletiellidae	Bourletiellinae	NC039558.1	Italy	Leo et al. [37]
7	*Bourletiella hortensis*	Symphypleona	Appendiciphora	Sminthuroidea	Bourletiellidae	Bourletiellinae	SRR17308033	NA	Collins et al. [36]
8	*Deuterosminthurus bicinctus*	Symphypleona	Appendiciphora	Sminthuroidea	Bourletiellidae	Parabourletiellinae	SRR21208389	NA	Collins et al. [36]
9	*Dicyrtoma fusca*	Symphypleona	Appendiciphora	Dicyrtomoidea	Dicyrtomidae	Dicyrtominae	SRR22586363	NA	Collins et al. [36]
10	*Dicyrtomina minuta*	Symphypleona	Appendiciphora	Dicyrtomoidea	Dicyrtomidae	Dicyrtominae	SRR22586362	NA	Collins et al. [36]
11	*Dicyrtomina saundersi*	Symphypleona	Appendiciphora	Dicyrtomoidea	Dicyrtomidae	Dicyrtominae	NC044134.1	Italy	Leo et al. [38]
12	*Lipothrix lubbocki*	Symphypleona	Appendiciphora	Sminthuroidea	Sminthuridae	Sphyrothecinae	MK431899.1	France	Sun et al. [39]
13	***Papirioides caishijiensis***	**Symphypleona**	**Appendiciphora**	**Dicyrtomoidea**	**Dicyrtomidae**	**Ptenothricinae**	**PQ035987.1**	**China**	**This study**
14	*Pseudobourletiella spinata*	Symphypleona	Appendiciphora	Sminthuroidea	Bourletiellidae	Bourletiellinae	SRR9066814	China	NP
15	*Ptenothrix huangshanensis*	Symphypleona	Appendiciphora	Dicyrtomoidea	Dicyrtomidae	Ptenothricinae	MK423965.1	China	Sun et al. [39]
16	*Sminthurides aquaticus*	Symphypleona	Sminthuridida	Sminthuridoidea	Sminthurididae	ND	OD987621.1	France	Schneider et al. [40]
17	*Sminthurides bifidus*	Symphypleona	Sminthuridida	Sminthuridoidea	Sminthurididae	ND	MK423964.1	China	Sun et al. [39]
18	*Sminthurinus aureous*	Symphypleona	Appendiciphora	Katiannoidea	Katiannidae	ND	SRR22586355	NA	Collins et al. [36]
19	*Sminthurinus bimaculatus*	Symphypleona	Appendiciphora	Katiannoidea	Katiannidae	ND	SRR22812166	Europe	NP
20	***Sminthurinus bimaculatus***	**Symphypleona**	**Appendiciphora**	**Katiannoidea**	**Katiannidae**	**ND**	**PQ035988.1**	**China**	**This study**
21	*Sminthurinus signatus*	Symphypleona	Appendiciphora	Katiannoidea	Katiannidae	ND	SRR17308055	NA	Collins et al. [36]
22	*Sminthurinus elegans*	Symphypleona	Appendiciphora	Katiannoidea	Katiannidae	ND	SRR22764688	NA	NP
23	*Sminthurus viridis*	Symphypleona	Appendiciphora	Sminthuroidea	Sminthuridae	Sminthurinae	EU016192.1	Germany	Carapelli et al. [41]
24	*Spatulosminthurus flaviceps*	Symphypleona	Appendiciphora	Sminthuroidea	Sminthuridae	Sminthurinae	SRR17308044	NA	Collins et al. [36]

**Table 2:** Lengths (in µm) for body parts of type specimens of *Arrhopalites beijingensis* sp. nov.

Body Part	*Arrhopalites beijingensis* sp. nov. (7 Females)			
	Holotype	Min	Max	Mean
Total (without appendages)	1000	700	1300	961
Head	300	250	440	330
Body (without head)	740	500	900	681
Head dorsum longest spine	17.5	15.0	25.0	19.7
Eye diameter	7.5	7.4	7.6	7.5
Antenna	625	473	865	627
Ant I	37.5	32.0	55.0	40.3
Ant II	87.5	70.0	127.5	91.6
Ant III	125.0	106.3	182.5	133.8
Ant IV	375.0	257.5	500.0	364.1
Ant III organ rods	7.5	4.7	10.0	6.4
Tibiotarsus I	200	150	265	198.7
Tibiotarsus II	205	145	255	204.1
Tibiotarsus III	235	175	350	250.1
Unguis I	52.5	40.0	67.5	50.6
Unguis II	50.0	40.0	62.5	47.9
Unguis III	42.5	37.5	55.0	43.4
Unguiculus I	30.0	25.0	37.5	30.7
Unguiculus II	25.0	20.0	32.5	24.6
Unguiculus III	25.0	20.0	32.5	24.5
Th II chaeta **m1**	12.5	12.5	20.0	16.3
Th II sensillum **a**	12.5	10.0	12.5	11.9
Th III sensillum **a**	12.5	10.0	12.5	11.7
Trichobothria **AB** distance	90.0	82.5	90.0	86.7
Trichobothria **BC** distance	60.0	55.0	62.5	59.2
Abd IV longest chaeta	30.0	30.0	61.0	46.7
Abd VI longest chaeta	50.0	42.5	62.5	51.7
Subanal appendages	25.0	25.0	31.7	27.0
Manubrium	175.0	150.0	200.0	174.9
Dens	187.5	161.0	270.0	195.8
Mucro	107.5	95.0	150.0	114.9
Dens spine **Ia**: length	15.0	15.0	25.7	19.7
Dens spine **Ia**: width	5.0	2.5	5.0	4.6
Dens spine **Ie**: length	15.0	12.5	27.5	17.2
Dens spine **Ie**: width	5.0	2.5	5.0	4.0

**Table 3:** Proportions for selected body parts of type specimens of *Arrhopalites beijingensis* sp. nov.

Ratio	*Arrhopalites beijingensis* sp. nov. (7 Females)			
	Holotype	Min	Max	Mean
Antenna/head	2.08	1.52	2.08	1.90
Ant IV/head	1.25	0.86	1.25	1.19
Ant II/Ant I	2.33	1.87	2.59	2.28
Ant III/Ant I	3.33	2.87	3.50	3.32
Ant IV/Ant I	10.00	6.87	10.00	9.01
Head/tibiotarsus I	1.50	1.50	1.85	1.66
Tibiotarsus II/I	1.03	0.96	1.11	1.01
Tibiotarsus III/I	1.18	1.17	1.43	1.26
Tibiotarsus I/unguis I	3.81	3.75	4.53	4.01
Tibiotarsus II/unguis II	4.10	3.63	4.75	4.27
Tibiotarsus III/unguis III	5.53	4.38	6.67	5.74
Unguis I/unguiculus I	1.75	1.46	1.80	1.64
Unguis II/unguiculus II	2.00	1.70	2.13	1.96
Unguis III/unguiculus III	1.70	1.63	2.11	1.78
Dens/mucro	1.74	1.61	1.80	1.70
Trichobothria: **AB**/**BC** distance	1.50	1.32	1.59	1.47
Abd IV **dI-1**/Th II **m1** chaeta	2.40	1.71	4.40	2.79
Abd IV/Abd VI longest chaeta	0.60	0.60	1.29	0.88
Abd IV **dI-1**/unguis III	1.18	0.89	1.47	1.18
Body total/tibiotarsus III	4.26	3.51	4.26	3.87
Body total/unguis I	19.05	17.50	20.64	19.21

## Data Availability

Data underlying this article are available in the GenBank Nucleotide and SRA databases at https://www.ncbi.nlm.nih.gov/ accessed on 15 December 2024. They can be accessed with Bioproject number PRJNA1125626; the accession numbers are as follows: *Arrhopalites beijingensis* sp. nov. mitogenome: PQ046244, SRA: SRR29636495; *Papirioides caishijiensis* mitogenome: PQ035987, SRA: SRR29636497; *Sminthurinus bimaculatus* mitogenome: PQ035988, SRA: SRR29636496. The sequences of the 11 mitogenomes assembled from raw SRA data were deposited in Figshare https://doi.org/10.6084/m9.figshare.26693296 accessed on 20 December 2024.

## Supplementary Materials

The following supporting information can be downloaded at https://www.mdpi.com/article/10.3390/insects16030314/s1: Table S1: Details about partitions and models used for the maximum likelihood inference with IQ-TREE. (Infor. = informative sites; Invar. = invariant sites). Table S2: Order, size, and position of mitochondrial protein-coding genes, ribosomal RNAs, and transfer RNAs of *Arrhopalites beijingensis* sp. nov. Table S3: Order, size, and position of mitochondrial protein-coding genes, ribosomal RNAs, and transfer RNAs of *Papirioides caishijiensis*. Table S4: Order, size, and position of mitochondrial protein-coding genes, ribosomal RNAs, and transfer RNAs of *Sminthurinus bimaculatus*. Figure S1: Circular representation of the mitogenome of *Arrhopalites beijingensis* sp. nov. The innermost circle shows the GC content; the middle circle shows the reads coverage, and the outermost circle shows the gene features, rRNA (pink), tRNA (purple), and CDS (green). Photo in the center represents the original coloration of a live specimen (photo by Yang HC and Zhou DK). Figure S2: Circular representation of the mitogenome of *Papirioides caishijiensis*. The innermost circle shows the GC content; the middle circle shows the reads coverage, and the outermost circle shows the gene features, rRNA (pink), tRNA (purple), and CDS (green). Photo in the center represents the original coloration of a live specimen (photo by Cheng, H.-J.). Figure S3: Circular representation of the mitogenome of *Sminthurinus bimaculatus*. The innermost circle shows the GC content; the middle circle shows the reads coverage, and the outermost circle shows the gene features, rRNA (pink), tRNA (purple), and CDS (green). Photo in the center represents the original coloration of a live specimen (photo by Cheng, H.-J.).
