# Supplementary material for: The First Cavernicolous Species of Arrhopalites (Collembola, Symphypleona, Arrhopalitidae) from China and Its Phylogenetic Position
Source: Insects. 2025 Mar 18;16(3):314. doi: 10.3390/insects16030314 (PMC11942825; doi:10.3390/insects16030314)
Supplement: Supplementary file 1 [file insects-16-00314-s001.zip › insects-3516483-supplementary.pdf]

Table S1. Details about partitions and models used for the maximum likelihood inference with IQTree. (Infor. = informative sites; Invar. = invariant sites).

Subset	Seqs	Interval	Sites	Infor.	Invar.	Model
ATP6	24	1-224	224	151	56	mtZOA+F+I+G4
ATP8	20	225-276	52	44	6	mtInv+I+G4
COX1	24	277-786	510	161	307	mtART+I+G4
COX2	24	787-1012	226	122	67	mtART+G4
COX3	24	1013-1273	261	140	90	mtART+I+G4
CYTB	24	1274-1640	367	180	127	mtART+I+G4
ND1	21	1641-1942	302	183	70	mtInv+G4
ND2	24	1943-2230	288	245	26	mtZOA+F+I+G4
ND3	24	2231-2344	114	80	29	mtART+I+G4
ND4	24	2345-2744	400	305	64	mtInv+F+I+G4
ND4L	24	2745-2829	85	68	9	mtInv+G4
ND5	24	2830-3381	552	384	117	mtInv+F+I+G4
ND6	24	3382-3530	149	128	9	mtInv+F+G4

Table S2. Order, size and position of mitochondrial protein-coding genes, ribosomal RNAs and transfer RNAs of *Arrhopalites beijingensis* **sp. nov.**

Start	End	Length (bp)	Direction	Type	Gene name	Gene product
128	1047	920	-	CDS	ND1	NADH dehydrogenase subunit 1
1141	1202	62	-	tRNA	trnL1(uag)	tRNA-Leu
1179	2417	1238	-	rRNA	l-rRNA	16S ribosomal RNA
2386	2446	61	-	tRNA	trnV(uac)	tRNA-Val
2442	3178	737	-	rRNA	s-rRNA	12S ribosomal RNA
3178	3240	62	+	tRNA	trnM(gca)	tRNA-Met
3262	4240	979	+	CDS	ND2	NADH dehydrogenase subunit 2
4240	4302	62	+	tRNA	trnW(uca)	tRNA-Trp
4318	4378	61	-	tRNA	trnY(gua)	tRNA-Tyr
4379	5903	1525	+	CDS	COX1	cytochrome c oxidase subunit I
5903	5964	62	+	tRNA	trnL(uaa)	tRNA-Leu
5963	6641	679	+	CDS	COX2	cytochrome c oxidase subunit II
6642	6705	64	+	tRNA	trnK(cuu)	tRNA-Lys
6704	6765	62	+	tRNA	trnD(guc)	tRNA-Asp
6765	6921	157	+	CDS	ATP8	ATP synthase F0 subunit 8
6914	7586	673	+	CDS	ATP6	ATP synthase F0 subunit 6
7593	8379	787	+	CDS	COX3	cytochrome c oxidase subunit III
8385	8446	62	+	tRNA	trnG(ucc)	tRNA-Gly
8446	8791	346	+	CDS	ND3	NADH dehydrogenase subunit 3
8798	8856	59	+	tRNA	trnA(ugc)	tRNA-Ala
8856	8918	62	+	tRNA	trnR(ucg)	tRNA-Arg
8918	8972	55	+	tRNA	trnS(gcu)	tRNA-Ser
8974	9036	63	+	tRNA	trnE(uuc)	tRNA-Glu
9034	9098	65	-	tRNA	trnF(gaa)	tRNA-Phe
9107	9170	64	+	tRNA	trnN(guu)	tRNA-Asn
9189	10875	1687	-	CDS	ND5	NADH dehydrogenase subunit 5
10875	10936	62	-	tRNA	trnH(gug)	tRNA-His
10910	12257	1348	-	CDS	ND4	NADH dehydrogenase subunit 4
12250	12532	283	-	CDS	ND4L	NADH dehydrogenase subunit 4L
12524	12587	64	+	tRNA	trnT(ugu)	tRNA-Thr
12589	12654	66	-	tRNA	trnP(ugg)	tRNA-Pro
12634	13102	469	+	CDS	ND6	NADH dehydrogenase subunit 6
13101	14232	1132	+	CDS	CYTB	cytochrome b
14233	14298	66	+	tRNA	trnS(uga)	tRNA-Ser

Table S3. Order, size and position of mitochondrial protein-coding genes, ribosomal RNAs and transfer RNAs of *Papirioides caishijiensis.*

Start	End	Length (bp)	Direction	Type	Gene name	Gene product
0	314	314				Control region
315	383	69	-	tRNA	trnQ(uug)	tRNA-Gln
381	446	66	+	tRNA	trnM(cau)	tRNA-Met
446	1436	991	+	CDS	ND2	NADH dehydrogenase subunit 2
1434	1502	69	+	tRNA	trnW(uca)	tRNA-Trp
1502	1565	64	-	tRNA	trnC(gca)	tRNA-Cys
1559	1622	64	-	tRNA	trnY(gua)	tRNA-Tyr
1637	1703	67	+	tRNA	trnI(gau)	tRNA-Ile
1733	3269	1537	+	CDS	COX1	cytochrome c oxidase subunit I
3264	3330	67	+	tRNA	trnL1(uaa)	tRNA-Leu1
3330	4014	685	+	CDS	COX2	cytochrome c oxidase subunit II
4015	4083	69	+	tRNA	trnK(cuu)	tRNA-Lys
4083	4146	64	+	tRNA	trnD(guc)	tRNA-Asp
4146	4308	163	+	CDS	ATP8	ATP synthase F0 subunit 8
4301	4973	673	+	CDS	ATP6	ATP synthase F0 subunit 6
4978	5767	790	+	CDS	COX3	cytochrome c oxidase subunit III
5770	5833	64	+	tRNA	trnG(ucc)	tRNA-Gly
5830	6178	349	+	CDS	ND3	NADH dehydrogenase subunit 3
6189	6253	65	+	tRNA	trnA(ugc)	tRNA-Ala
6253	6315	63	+	tRNA	trnR(ucg)	tRNA-Arg
6315	6378	64	+	tRNA	trnN(guu)	tRNA-Asn
6379	6446	68	+	tRNA	trnS(gcu)	tRNA-Ser
6445	6508	64	+	tRNA	trnE(uuc)	tRNA-Glu
6508	6571	64	-	tRNA	trnF(gaa)	tRNA-Phe
6568	8218	1650	-	CDS	ND5	NADH dehydrogenase subunit 5
8261	8324	64	-	tRNA	trnH(gug)	tRNA-His
8328	9663	1336	-	CDS	ND4	NADH dehydrogenase subunit 4
9662	9932	271	-	CDS	ND4L	NADH dehydrogenase subunit 4L
9934	9999	66	+	tRNA	trnT(ugu)	tRNA-Thr
9999	10064	66	-	tRNA	trnP(ugg)	tRNA-Pro
10065	10536	472	+	CDS	ND6	NADH dehydrogenase subunit 6
10535	11663	1129	+	CDS	CYTB	cytochrome b
11661	11733	73	+	tRNA	trnS(uga)	tRNA-Ser
11753	12689	937	-	CDS	ND1	NADH dehydrogenase subunit 1
12702	14021	1320	-	rRNA	l-rRNA	16S ribosomal RNA
13986	14053	68	-	tRNA	trnV(uac)	tRNA-Val
14052	14800	749	-	rRNA	s-rRNA	12S ribosomal RNA
14801	15271	470				Control region

Table S4. Order, size and position of mitochondrial protein-coding genes, ribosomal RNAs and transfer RNAs of *Sminthurinus bimaculatus.*

Start	End	Length (bp)	Direction	Type	Gene name
0	335	335			Control region
336	398	63	+	tRNA	trnI(gau)
394	460	67	-	tRNA	trnQ(uug)
458	526	69	+	tRNA	trnM(cau)
526	1525	1000	+	CDS	ND2
1526	1589	64	+	tRNA	trnW(uca)
1587	1656	70	-	tRNA	trnC(gca)
1656	1723	68	-	tRNA	trnY(gua)
1705	3259	1555	+	CDS	COX1
3254	3316	63	+	tRNA	trnL1(uaa)
3316	4024	709	+	CDS	COX2
4001	4068	68	+	tRNA	trnK(cuu)
4067	4130	64	+	tRNA	trnD(guc)
4131	4284	153	+	CDS	ATP8
4282	4960	679	+	CDS	ATP6
4965	5754	790	+	CDS	COX3
5754	5815	62	+	tRNA	trnG(ucc)
5803	6155	353	+	CDS	ND3
6155	6215	61	+	tRNA	trnR(ucg)
6217	6280	64	+	tRNA	trnA(ugc)
6279	6342	64	+	tRNA	trnN(guu)
6341	6408	68	+	tRNA	trnS(gcu)
6409	6473	65	+	tRNA	trnE(uuc)
6484	6549	66	-	tRNA	trnF(gaa)
6523	8251	1729	-	CDS	ND5
8274	8336	63	-	tRNA	trnH(gug)
8348	9683	1336	-	CDS	ND4
9707	9983	277	-	CDS	ND4L
9975	10040	66	+	tRNA	trnT(ugu)
10037	10100	64	-	tRNA	trnP(ugg)
10101	10577	477	+	CDS	ND6
10570	11707	1138	+	CDS	CYTB
11705	11776	72	+	tRNA	trnS(uga)
11819	12752	934	-	CDS	ND1
12752	12819	68	-	tRNA	trnL2(uag)
12769	14052	1284	-	rRNA	l-rRNA
14020	14085	66	-	tRNA	trnV(uac)
14082	14823	742	-	rRNA	s-rRNA
14823	14922	99			Control region

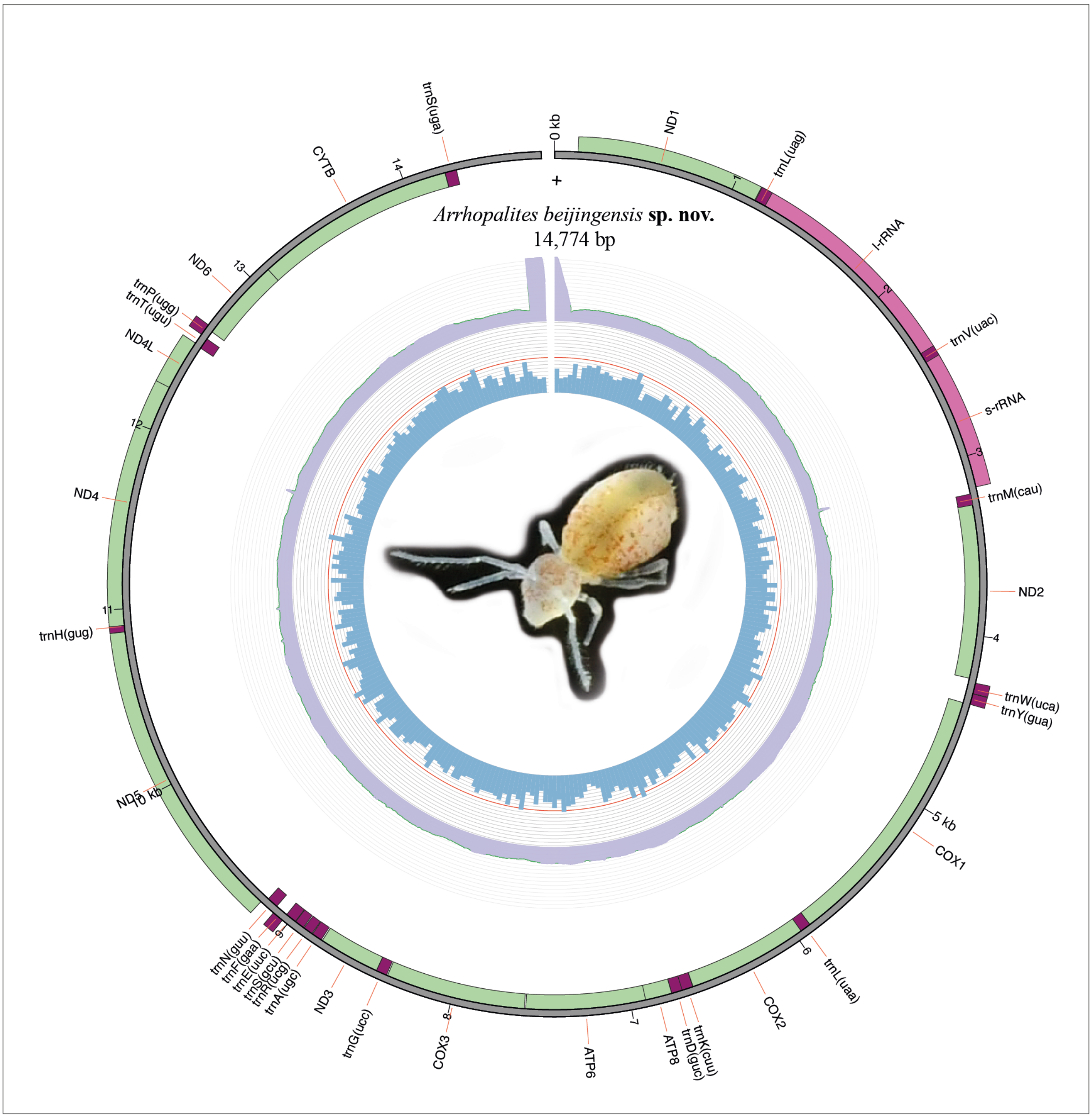

Figure S1. Circular representation of the mitogenome of *Arrhopalites beijingensis* **sp. nov.** The innermost circle shows the GC content; the middle circle shows the reads coverage, and the outermost circle shows the gene features, rRNA (pink), tRNA (purple), and CDS (green). Photo in the center represents the original coloration of a live specimen (Photo by: Yang HC and Zhou DK).

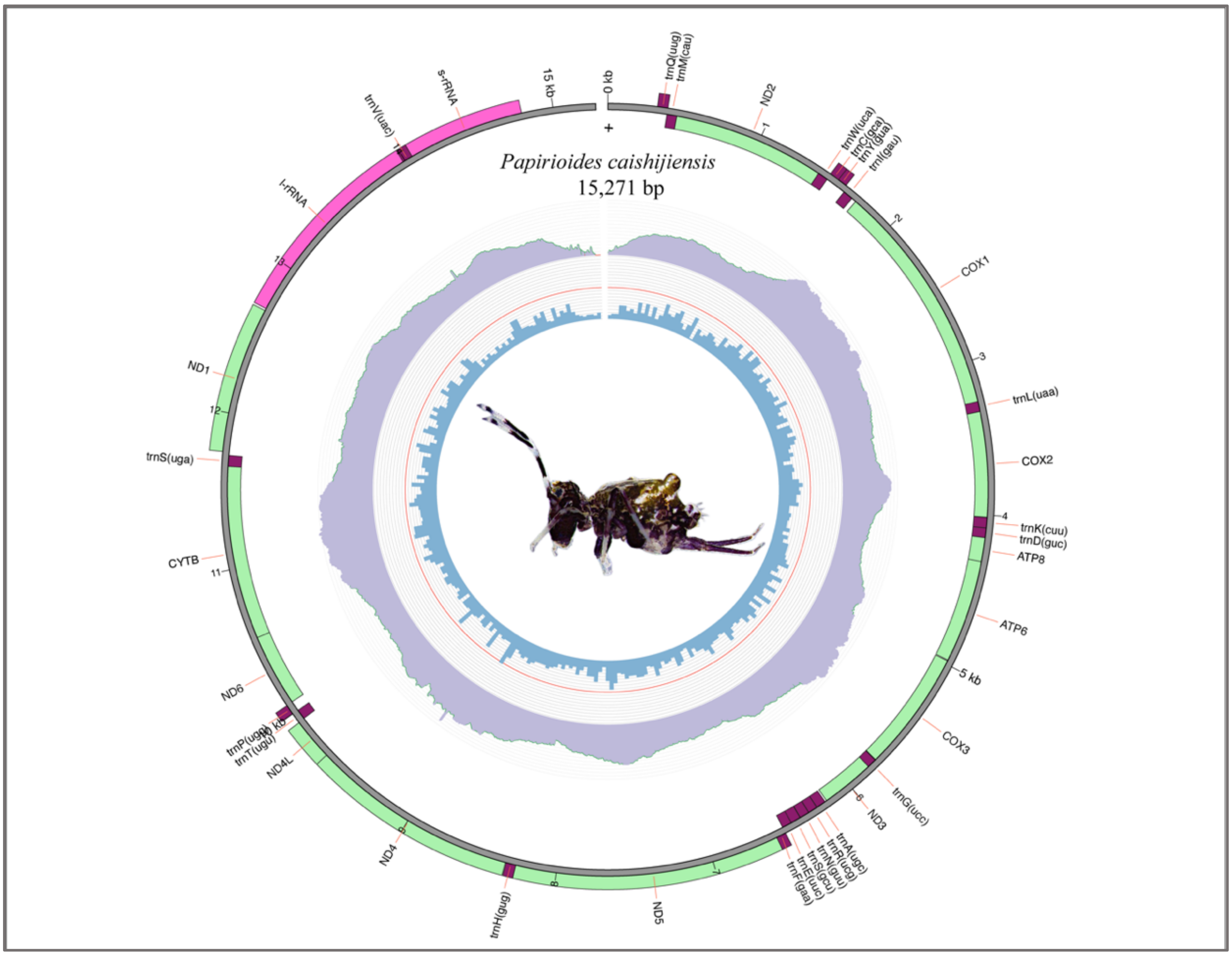

Figure S2. Circular representation of the mitogenome of *Papirioides caishijiensis*. The innermost circle shows the GC content; the middle circle shows the reads coverage, and the outermost circle shows the gene features, rRNA (pink), tRNA (purple), and CDS (green). Photo in the center represents the original coloration of a live specimen (Photo by: Cheng, H.-J.).

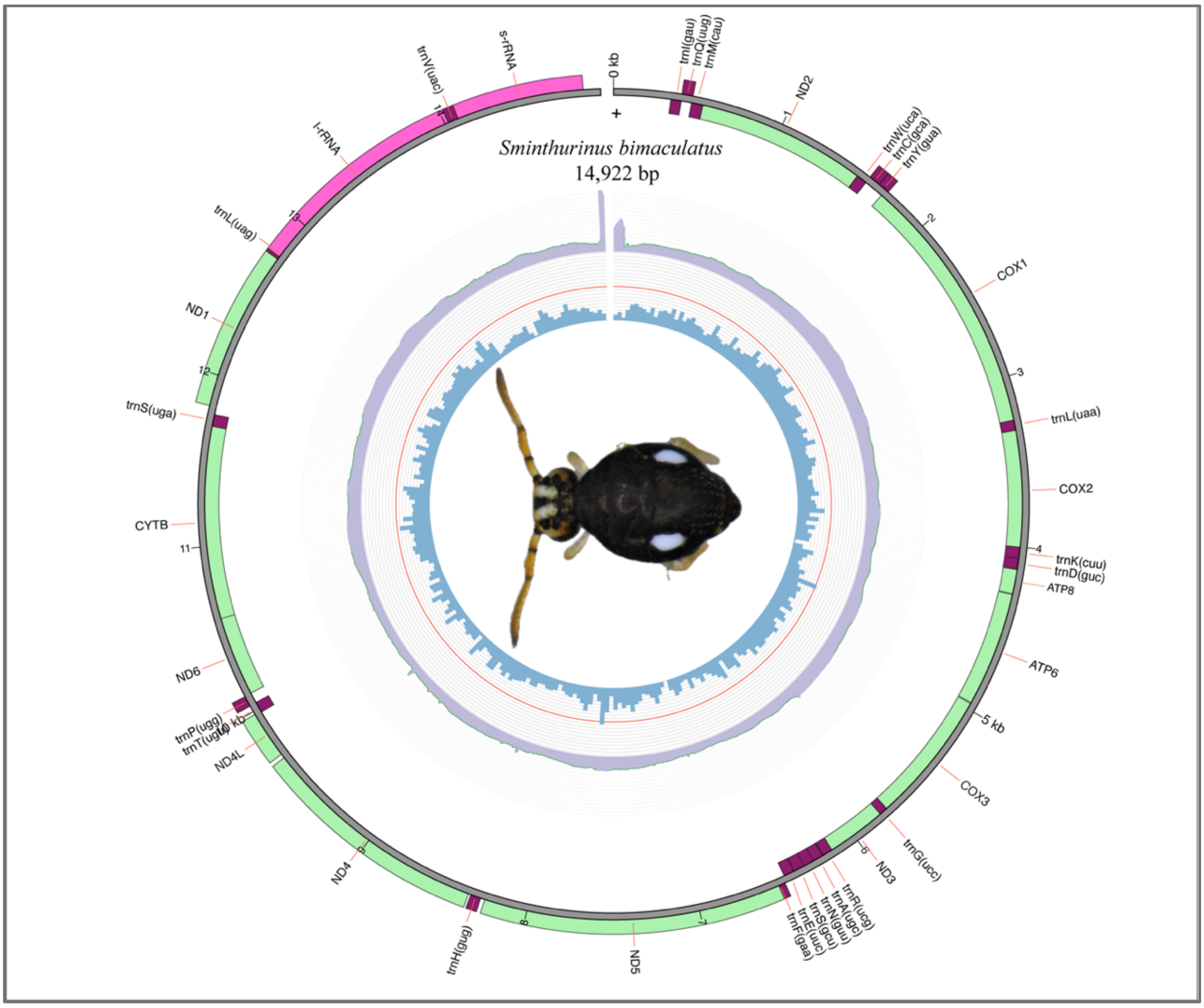

Figure S3. Circular representation of the mitogenome of *Sminthurinus bimaculatus*. The innermost circle shows the GC content; the middle circle shows the reads coverage, and the outermost circle shows the gene features, rRNA (pink), tRNA (purple), and CDS (green). Photo in the center represents the original coloration of a live specimen (Photo by: Cheng, H.-J.).
